# miRNA-432 and SLC38A1 as Predictors of Hepatocellular Carcinoma Complicated with Alcoholic Steatohepatitis

**DOI:** 10.1155/2022/4832611

**Published:** 2022-05-25

**Authors:** Chao Cai, Jing Lin, Ji Li, Xiao-Dong Wang, Lan-Man Xu, Da-Zhi Chen, Yong-Ping Chen

**Affiliations:** ^1^Department of Infectious Diseases, The First Affiliated Hospital of Wenzhou Medical University, Hepatology Institute of Wenzhou Medical University, Zhejiang Key Laboratory of Hepatology, Wenzhou, 325000 Zhejiang, China; ^2^Department of Infectious Diseases and Liver Diseases, Ningbo Medical Center Lihuili Eastern Hospital, Ningbo, 315040 Zhejiang, China; ^3^Department of Infectious Diseases and Liver Diseases, Taipei Medical University Ningbo Medical Center, Ningbo, 315040 Zhejiang, China; ^4^Department of Gastroenterology, The First Hospital of Peking University, Beijing 100034, China

## Abstract

Alcoholic steatohepatitis (ASH) is asymptomatic in the early stages and is typically advanced at the time of diagnosis. With the global rise in alcohol abuse, ASH is currently among the most detrimental diseases around the world. Hepatocellular carcinoma (HCC) is one of the final outcomes of numerous liver diseases. However, at present, HCC screening is mostly focused on liver cancer development. Moreover, there is no effective biomarker to predict the prognosis and recurrence of liver cancer. Meanwhile, there are limited studies on the prognosis and recurrence of HCC patients complicated with ASH. In this study, using bioinformatic analysis as well as cellular and animal models, we screened the differentially expressed (DE) miRNA-432 and SLC38A1 gene in ASH. Based on our analysis, miRNA-432 targeted SLC38A1, and the levels of miRNA-432 and SLC38A1 could accurately predict the overall survival (OS) and relapse free survival (RFS) in patients with liver cancer. Hence, these two genetic elements have the potential to synergistically predict the prognosis and recurrence of HCC complicated with ASH.

## 1. Introduction

Alcoholic liver disease (ALD) is a hepatic disorder caused by chronic or excessive alcohol ingestion. It is characterized by the alcoholic fatty liver (AFL), which eventually progresses to alcoholic steatohepatitis (ASH). Chronic ASH, manifested by hepatic inflammation, can then lead to alcohol-induced hepatic fibrosis (AHF), alcoholic cirrhosis (AC), and even hepatocellular carcinoma (HCC) [1]. From1980 to 2016, the Global Burden of Disease (GBD) project estimated approximately 334,900 (27%) chronic ALD-induced deaths from cirrhosis and chronic liver disease in the global. In addition, 245,000 people died from alcohol-related HCC, accounting for 30% of all HCC deaths [2]. ASH is usually asymptomatic in the early stage and is already advanced at the time of diagnosis. It is a large contributor to mortality and morbidity and facilitates rapid progression to fibrosis and HCC [3]. Traditional biochemical index tests only monitor the presence of ASH or HCC. However, there is a lack of indicators that assess the survival and recurrence rates of HCC, caused by ASH at varying stages. Therefore, it is necessary to investigate the ASH-related molecular biomarkers to aid in the prevention and treatment of ASH-induced HCC.

MicroRNAs (miRNAs) are noncoding RNAs, consisting of 20-22 nucleotides in length, and they post transcriptionally and translationally modulate gene expression. ALD regulation via miRNAs is a hot topic in the research field today. Several studies demonstrated that miR-21, miR-233, miR-214, miR-192, and miR-155 modulate inflammation and oxidative stress in ASH [4–7]. Additionally, miR-34a and miR-122 accelerate the development of AFL [8, 9]. miRNAs have great potential as predictive biomarkers, as certain miRNAs are DE in ASH and can be detected in liver tissue via real-time quantitative PCR (RT-qPCR). However, it is possible that one miRNA can regulate multiple proteins. Therefore, it is crucial to determine the underlying mechanism of miRNAs' predictive function in ASH and establish whether the corresponding protein targets offer similar predictive functions in ASH patients.

Bioinformatics data mining can easily identify novel essential genes and noncoding RNAs related to different disease pathogenesis. This information provides valuable insights and targets for additional investigations. Therefore, little research has been reported on the ASH. Here, we extracted the mRNA and miRNA expression profiles (GSE59492, GSE28619, and GSE155907) from ASH patients from the Gene Expression Omnibus (GEO) database. In addition, we also performed DE, Gene Ontology (GO) functional annotation, and Kyoto Encyclopedia of Genes and Genomes (KEGG) pathway enrichment analysis. Next, we constructed putative miRNA–mRNA networks. To confirm the profile of these miRNAs and mRNAs, we conducted the RT-qPCR analysis *in vivo* and *in vitro*. Lastly, we employed the Human Protein Atlas (THPA) database to explore the significance of these DEs as prognostic bioindicators of ASH-induced HCC. This study provides a novel noninvasive approach for predicting the prognosis and recurrence of HCC complicated with ASH.

## 2. Materials and Methods

### 2.1. Microarray Information

To compare the expression profile of mRNA and miRNA in ASH versus healthy hepatic tissue, we retrieved this information from the NCBI-GEO (http://www.ncbi.nlm.nih.gov/geo) website, an open-access microarray profile and next-generation sequencing database. We only collected datasets involving human liver tissues. The collected datasets were then screened for information prior to evaluation. Finally, the following mRNA/miRNA expression profiles were selected: GSE28619, according to the GPL570 platform ([HG-U133_Plus_2] Affymetrix Human Genome U133 Plus 2.0 Array), which included 7 control liver specimens and 15 ASH liver specimens; GSE155907, according to the GPL21290 platform (Illumina HiSeq 3000), which included 4 control liver specimens and 5 ASH liver specimens; and GSE59492, according to the GPL16384 platform ([miRNA-3] Affymetrix Multispecies miRNA-3 Array), which included 6 control liver specimens and 13 ASH liver specimens.

### 2.2. DE gene (DEG) and miRNA (DE miRNA) Identification

We downloaded the series matrix file from the GEO website; then, we normalized the data with the normalize between array function of the R package “LIMMA” from the Bioconductor project [10]. The DEG identification was done with the “LIMMA” package in R (version 4.0.3). The *P* value <0.05 and |log2FC| > 1 were adjusted as the threshold criterion. Lastly, the common DEGs are presented in a Venn diagram.

### 2.3. Functional Annotation and Pathway Enrichment Analysis (FAPEA)

GO functional annotation and KEGG pathway enrichment analysis were used for DEGs' enrichment analysis. The enriched miRNA clusters, families, miRNA-related diseases, and DE miRNA functions were assessed via TAM 2.0 (http://www.lirmed.com/tam2/). *P* value <0.05 was adjusted as the significance threshold [11–13].

### 2.4. Protein-Protein Interaction (PPI) Axis and Modular Analyses

The DEG PPI axis was generated via the STRING database (http://string-db.org, version 11.0), and a combined score > 0.90 was deemed as the significance threshold [14]. Cytoscape (version 3.6.1) was employed to generate visual axes of molecular associations [15]. The Cytoscape plug-in Molecular Complex Detection (MCODE) (version 1.4.2) was used to identify closely associated modules from the PPI axis. Significant modular genes are graphically illustrated via the MCODE plug-in. The selection criteria were adjusted as follows: MCODE score > 5, node score threshold = 0.2, degree threshold = 2, *k* − score = 2, and max depth = 100. The DEGs of top molecules were enriched for KEGG enrichment pathway analysis using the ClueGO plug-in.

### 2.5. Target Gene Prediction of DE miRNAs

To predict DE miRNA targets, the miRTarBase (https://mircarta.cs.uni-saarland.de/targets_search/), microT-CDs (https://mircarta.cs.uni-saarland.de/targets_search/), TargetScan (http://www.targetscan.org/), miRDB (http://www.mirdb.org/), and miRWalk (http://mirwalk.uni-hd.de/) online databases were employed. Moreover, genes recognized by ≥4 databases were deemed as DE miRNA targets [16–20]. DEGs that intersected with estimated target genes were selected for additional investigation. Lastly, the Cytoscape software (version 3.6.1) was employed to construct regulatory axes between the DE miRNAs and selected DEGs.

### 2.6. Cell Culture

We acquired the AML12 cell line from the Institute of Biochemistry and Cell Biology of the Chinese Academy of Sciences (Shanghai, China) and maintained them in DMEM/F12 (Gibco, Thermo Fisher Scientific, Inc., Waltham, MA, USA), with 10% fetal bovine serum (FBS) (Gibco), 100 mg/mL streptomycin, and 100 U/mL penicillin (Invitrogen, Shanghai, China) in a humid chamber at 5% CO_2_ and 37°C. Once the cells reached 70-80% confluency, they were treated with 200 mM alcohol for 24 h.

### 2.7. Animal Experiments and Sample Collection

Twelve 6-week-old male wild-type (WT) C57BL/6 mice, with bodyweight around 18~20 g, were acquired from the Shanghai Laboratory Animal Center (Shanghai, China) to establish an experimental ASH mouse model and healthy controls. All mice were arbitrarily and equally separated into two populations. The control population (C, *n* = 6) received the Lieber-DeCali diet (Trophic Animal Feed High-tech Co., Jiangsu, China) for 7 weeks, whereas the ASH model mice (M, *n* = 6) first received the Lieber-DeCali diet for 2 weeks then received a ratio of the Lieber-Decarli and ethanol diets (5% ethanol) that went from 2 : 1 to 1 : 1 to 1 : 2 at days 2, 4, and 6, respectively, over 1 week; lastly, the Lieber-DeCali ethanol diet was provided for 4 weeks. At the end of the 7th week, ethanol (5 g/kg body weight, 40%) or isothermal dextrin (8.9 g/kg body weight) was administered to the model and control groups. After 10 hours of gavage, all mice were sacrificed and the blood and liver samples were collected. Our mouse protocols received ethical approval from the Wenzhou Medical University, and we strictly followed the guidelines of the Care and Use of Laboratory Animals.

### 2.8. Assessment of the Serum ALT and AST Levels

For serum collection, we centrifuged blood samples at 3500 rpm for 10 min and then measured the serum ALT and AST contents via an automated biochemical analyzer (Abbott Laboratories, Chicago, IL, United States).

### 2.9. Hepatic Histopathological Examination

Hepatic tissues underwent fixation in 4% paraformaldehyde, prior to paraffin embedding after routine processing. Next, we sliced the embedded tissues into 4 *μ*m sections and stained them with hematoxylin and eosin (H&E). We also rapidly froze unstained 4 *μ*m hepatic slices prior to staining with Oil Red O. Staining analysis was done via images from three arbitrary vision fields from each slide.

### 2.10. Real-Time Quantitative PCR (RT-qPCR)

Total RNA was isolated from the hepatic specimens and cultured cells, following directions from the RNA isolation kit (Aidlab Biotechnologies Co., Beijing, China). Next, we converted the isolated RNA into cDNA, following kit guidelines (Prime ScriptTM RT reagent kit, Takara, Otsu, Shiga, Japan), and RT-qPCR was carried out to measure the expression of SOX4, SLC38A1, SORI1, COL4A1, CCL20, SH3BGRL, SC5D, GFRA1, FXYD1, miR-432, miR-21a-3P, miR-21a-5P, miR-214-5P, miR-182-3P, and miR-132-5P using the TB Green Premix Ex Taq TM II (Takara) and the ABI7500 Fast real-time PCR system (Applied Biosystems, Life Technologies, Waltham, MA, United States). Relative gene levels were determined via the 2^−*ΔΔ*Ct^ formula and normalized to the internal control gene GAPDH or U6. The employed primer sequences are listed in Table 1.

### 2.11. Hub Gene Selection and Analysis

The Human Protein Atlas (THPA) (https://www.proteinatlas.org/), a database containing images of immunohistochemical- (IHC-) based tissue microarrays, was used for the verification of hub genes [21]. We also generated OS and RFS Kaplan-Meier curves using the Python package.

### 2.12. Statistics and Data Analysis

We employed the GraphPad Prism software (version 8.0) to analyze all data, and presented them as mean ± SD. Intergroup comparisons were assessed via the student's *t*-test. *P* < 0.05 was set as the significance threshold.

## 3. Results

### 3.1. Screening for DE miRNAs

**Figure 1:**
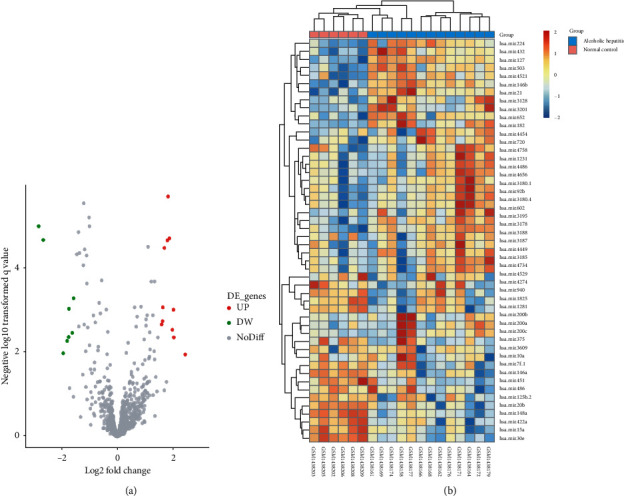
The screening of DE miRNAs. (a) The volcano plot of DE miRNAs between normal and ASH tissues in the GSE59492 profile datasets. (b) The heatmap of DE miRNA between normal and ASH tissues in the GSE59492 profile datasets.

19 DE miRNAs were acquired from the miRNA profile dataset GSE59492, including 11 miRNAs with enhanced expression and 8 miRNAs with diminished expression (Figure 1). The detailed data of DE miRNAs are provided in Table 2.

### 3.2. FAPEA

**Figure 2:**
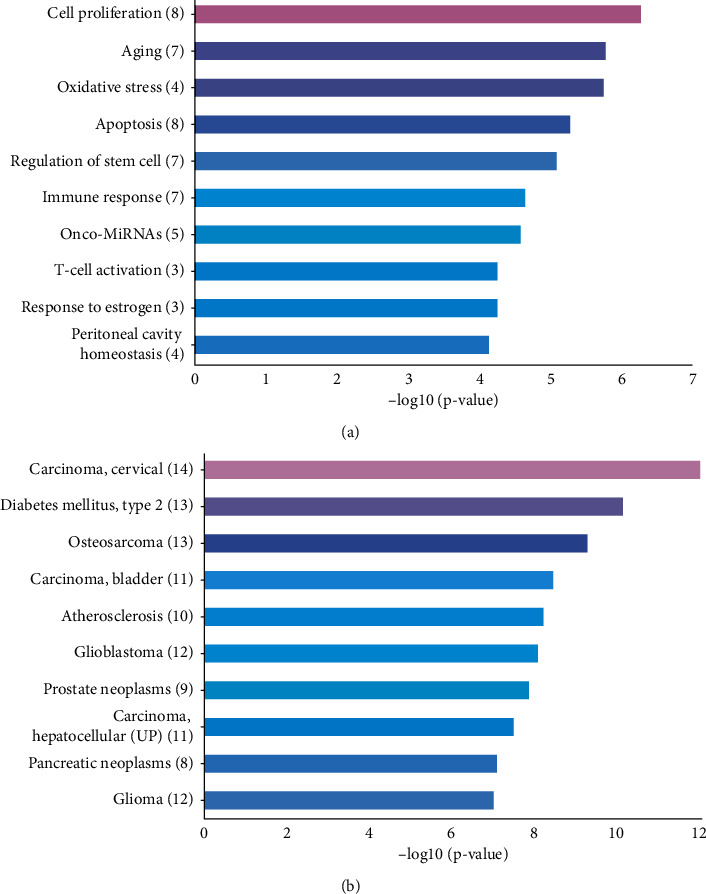
The functional annotation of DE miRNAs. (a) The 10 leading functional terms for DE miRNAs. (b) The 10 leading disease terms for DE miRNAs.

TAM 2.0 online was employed for DE miRNAs enrichment analysis. The DE miRNA was mainly enriched in cell proliferation, aging, and oxidative stress. Disease-related DE miRNA enrichment was mainly in carcinoma cancer, type 2 diabetes melilites, and osteosarcoma (Figure 2).

### 3.3. Comparison with the DE miRNA Expression Profile

**Figure 3:**
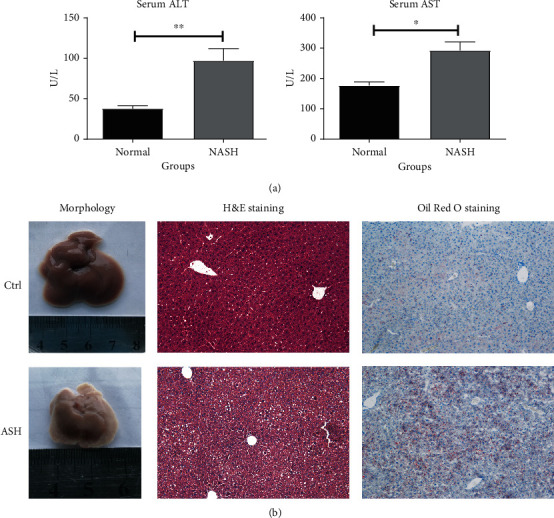
The liver biochemical analysis, morphology, HE, and Oil Red O staining. (a) An illustration of the serum ALT and AST levels in normal and ASH models. (b) Morphology, HE, and Oil Red O staining of liver tissue. ^∗^*P* < 0.05, ^∗∗^*P* < 0.01.

**Figure 4:**
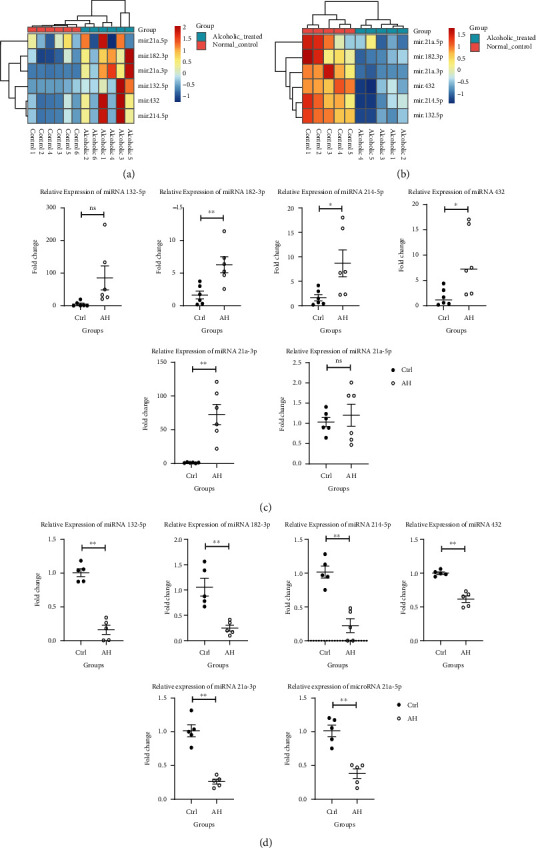
Comparing DE miRNA levels. (a) The heatmap of significant DE miRNA expressions between normal and ASH tissues in animal models. (b) The heatmap of significant miRNA expressions between normal and alcohol-stimulated cell models. (c) The relative microRNA levels, using RT-qPCR in animal models. (d) The relative microRNA levels, using RT-qPCR in cell models. ^∗^*P* < 0.05, ^∗∗^*P* < 0.01.

The DE miRNAs expression was validated both *in vivo* and *in vitro*. In the ASH animal models, serum ALT and AST levels were markedly elevated, compared to the control group. We also observed a dramatic decrease in liver sizes in ASH mice, as opposed to control mice. In addition, we evaluated liver histology to assess liver damage in mice. The liver tissues from ASH mice models also demonstrated significant damage and lipid deposition, relative to the control mice (Figure 3). The RT-qPCR results are presented in Figure 4. According to the RT-qPCR results, microRNA 182-3P, microRNA 214-5P, microRNA 432, and microRNA 21a-3P were highly expressed in the ASH model group, relative to controls. However, in cellular models, the expression profiles of these miRNAs were diminished (Figure 4).

### 3.4. Screening of DEGs

**Figure 5:**
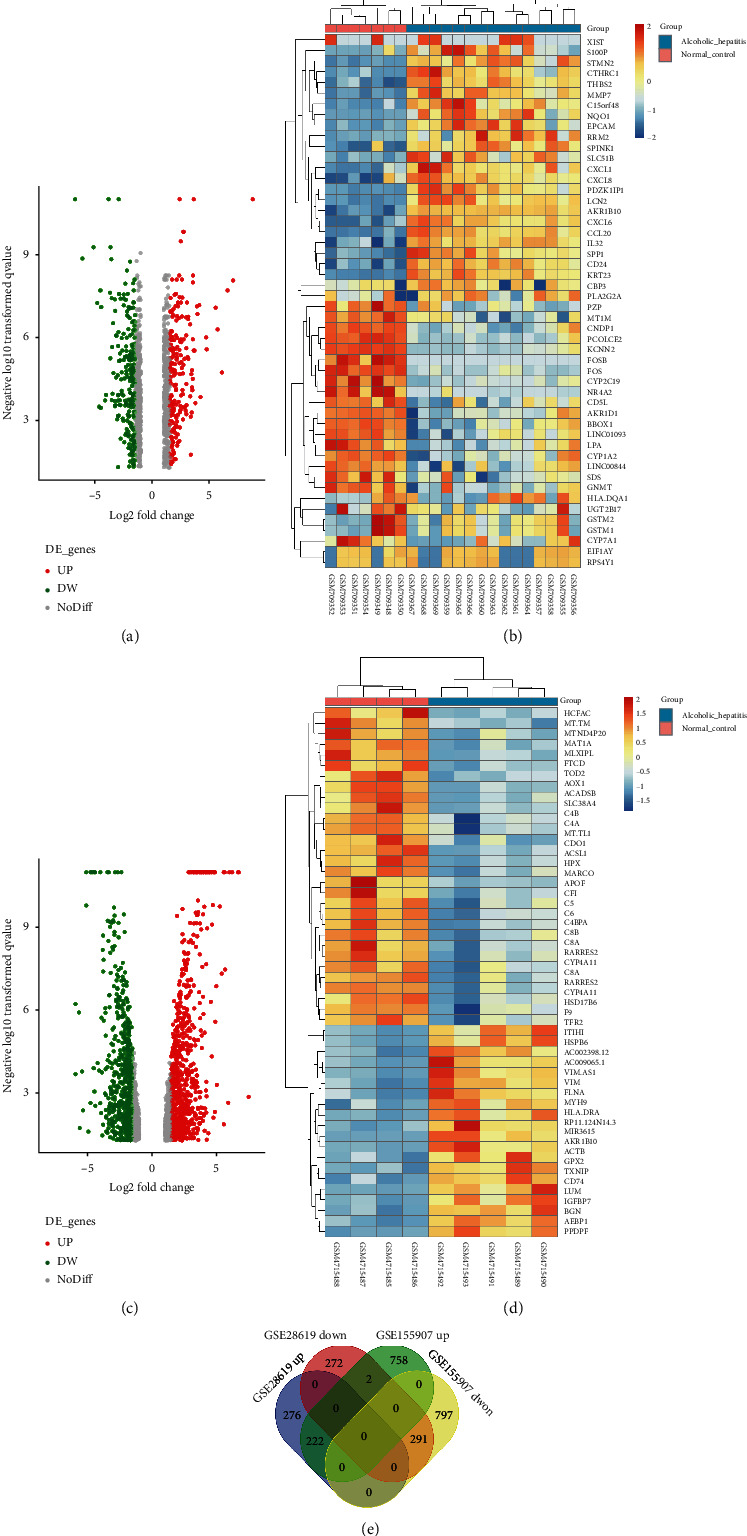
Screening of DEGs. (a) The volcano plot of DEGs between normal and ASH tissues in the GSE28619 profile datasets. (b) The heatmap of DEGs between normal and ASH tissues in the GSE28619 profile datasets. (c) The volcano plot of DEGs between normal and ASH tissues in the GSE155907 profile datasets. (d) The heatmap of DEGs between normal and ASH tissues in the GSE155907 profile datasets. (e) DEGs shared in the GSE28619 and GSE155907 profile datasets, as per the Venn diagram. DEGs: differentially expressed genes.

In total, there were 1064 and 2071 DEGs obtained from the GSE28619 and GSE155907 datasets. There were 222 identical elevated genes and 291 identical diminished genes in both datasets (Figure 5). List of consistent DEGs was provided in Table 3.

### 3.5. FAPEA

**Figure 6:**
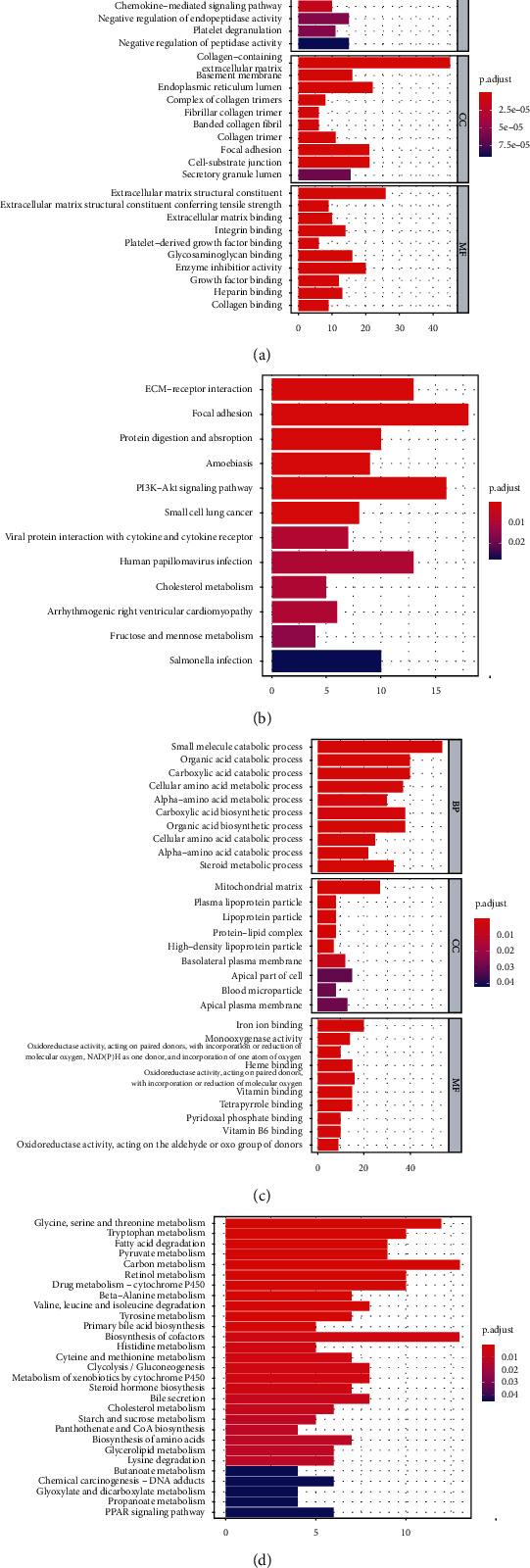
DEGs' GO and KEGG network enrichment analyses. (a) The 10 leading GO terms of highly expressed DEGs. (b) The KEGG analysis of highly expressed DEGs. (c) The 10 leading GO terms of scarcely expressed DEGs. (d) The KEGG analysis of scarcely expressed DEGs. GO: Gene Ontology; KEGG: Kyoto Encyclopedia of Genes and Genomes; DEGs: differentially expressed genes.

The GO terms consisted of several components, namely, cellular (CC), biological process (BP), and molecular function (MF). The 10 leading GO terms of elevated DEGs are depicted in Figure 6(a). The Elevated DEG enrichments result in BP were “extracellular matrix organization” and “extracellular structure organization.” DEG enrichment result in CC was “collagen-containing.” DEG enrichment in MF was “extracellular matrix structural.” Moreover, based on our KEGG analysis, elevated DEG enrichments were in the “focal adhesion” and the “PI3K-AKT network” (Figure 6(b)).

The 10 leading GO terms for diminished DEGs are depicted in Figure 6(c), and they included “small molecule catabolic process” in CC, “mitochondrial matrix” in BP, and “iron ion binding” in MF. Based on the KEGG analysis, diminished DEGs were mostly enriched in “glycine, serine, and threonine metabolism,” “carbon metabolism,” “biosynthesis of cofactors,” and “fatty acid degradation” (Figure 6(d)).

### 3.6. Identification of Hub Genes using DEGs PPI and Modular Analyses

**Figure 7:**
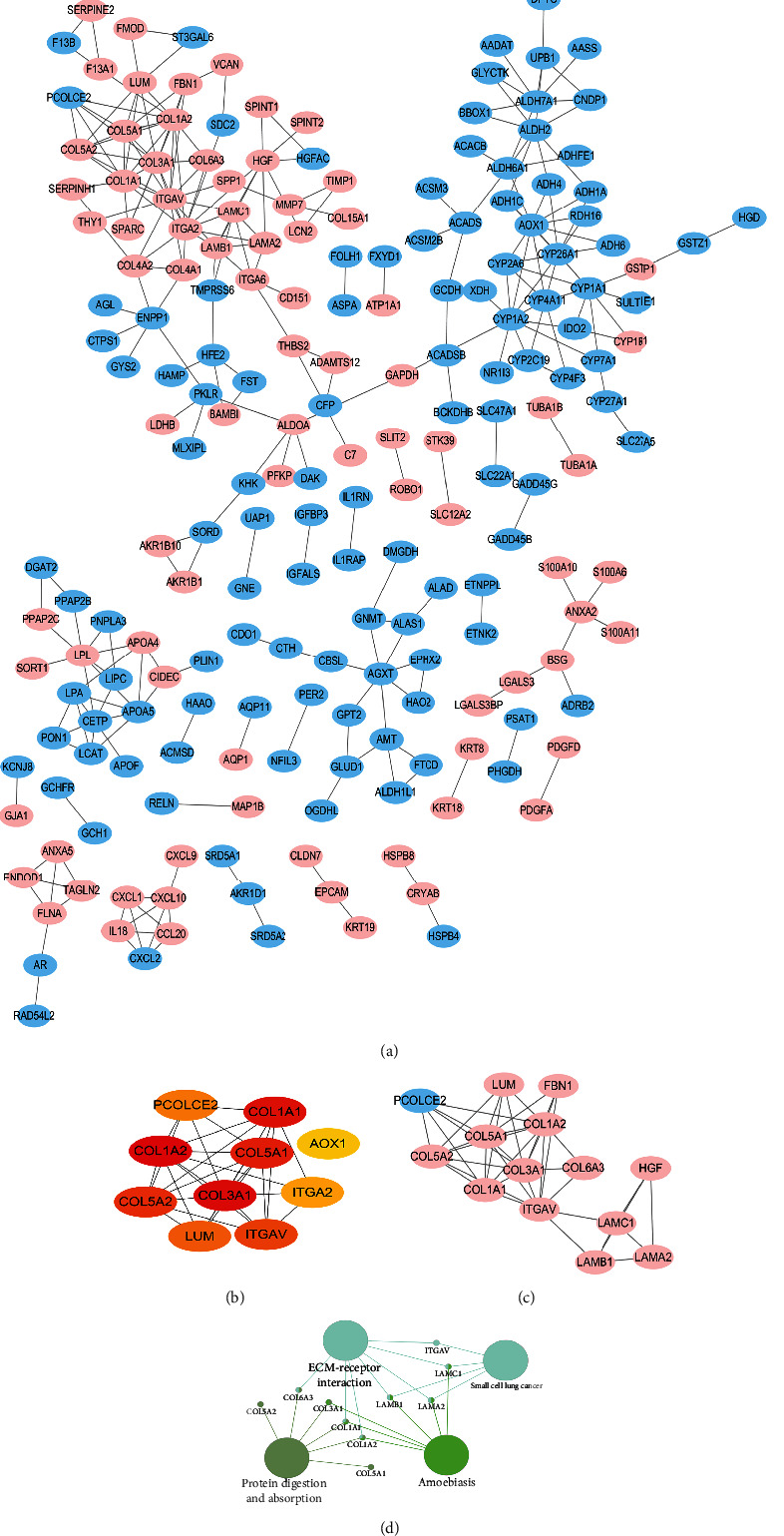
The PPI axis and modular analyses. (a) The DEG PPI axis. Upregulated DEGs were represented by red nodes, and downregulated DEGs by blue nodes. The lines indicated the regulatory relationship of DEGs. (b) The 10 leading hub genes with an elevated degree of connectivity. (c) The top module was identified by the PPI axis. (d) KEGG analysis of the top module. PPI: protein-protein interaction; DEGs: differentially expressed genes; KEGG: Kyoto Encyclopedia of Genes and Genomes.

We employed the STRING database and Cystoscope software to establish hub genes. Following filtering of the DEGs PPI network complex, we identified 204 DEGs with 280 edges (Figure 7(a)). Next, the 10 leading hub genes were recognized by screening for high levels of connectivity using the cytoHubba plug-in. The collagen (COL) family, including COL type I alpha 1 (COL1A1), COL type I alpha 2 (COL1A2), COL type III alpha 1 (COL3A1), COL type V alpha 1 (COL5A1), and COL type V alpha 2 (COL5A2), was primarily included (Figure 7(b)). Overall, 14 DEGs with 40 edges were obtained by screening for significant module degrees using the MCODE plug-in (Figure 7(c)). Lastly, using the KEGG analysis, we revealed that the top module DEG enrichments were in “ECM-receptor interaction” as well as “protein digestion and absorption,” using the ClueGO plug-in (Figure 7(d)).

### 3.7. DE miRNA-DEG Modulatory Axis Construction

**Figure 8:**
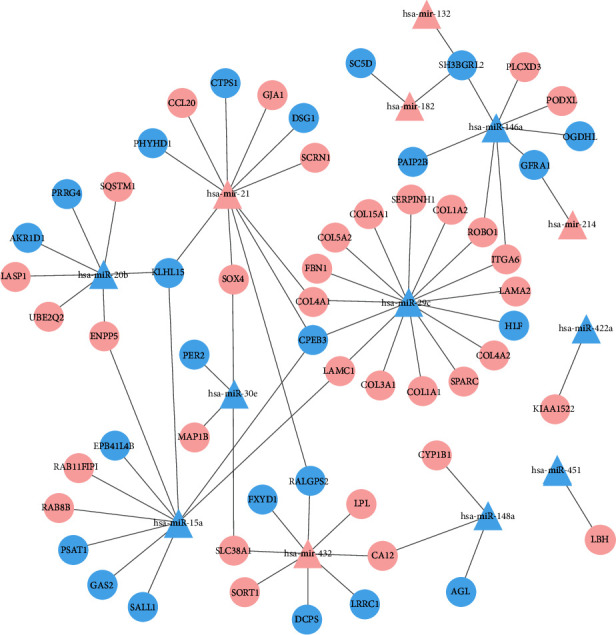
DE miRNA-DEG modulatory axis. The pink nodes represented the highly expressed DEGs and DE miRNAs, and the blue nodes represented the scarcely expressed DEGs and DE miRNAs. The lines indicated the modulatory association between DE miRNAs and DEGs. DE: differentially expressed; miRNAs: microRNAs; DEGs: differentially expressed genes.

We estimated DE miRNA target genes from online databases, using estimated target genes and DEGs. Overall, we identified 57 target DEGs and developed a new DE miRNA-DEG modulatory axis in ASH, including microRNA 432-SLC38A1 (Figure 8).

### 3.8. Comparing the Expression Profiles of Target Genes

**Figure 9:**
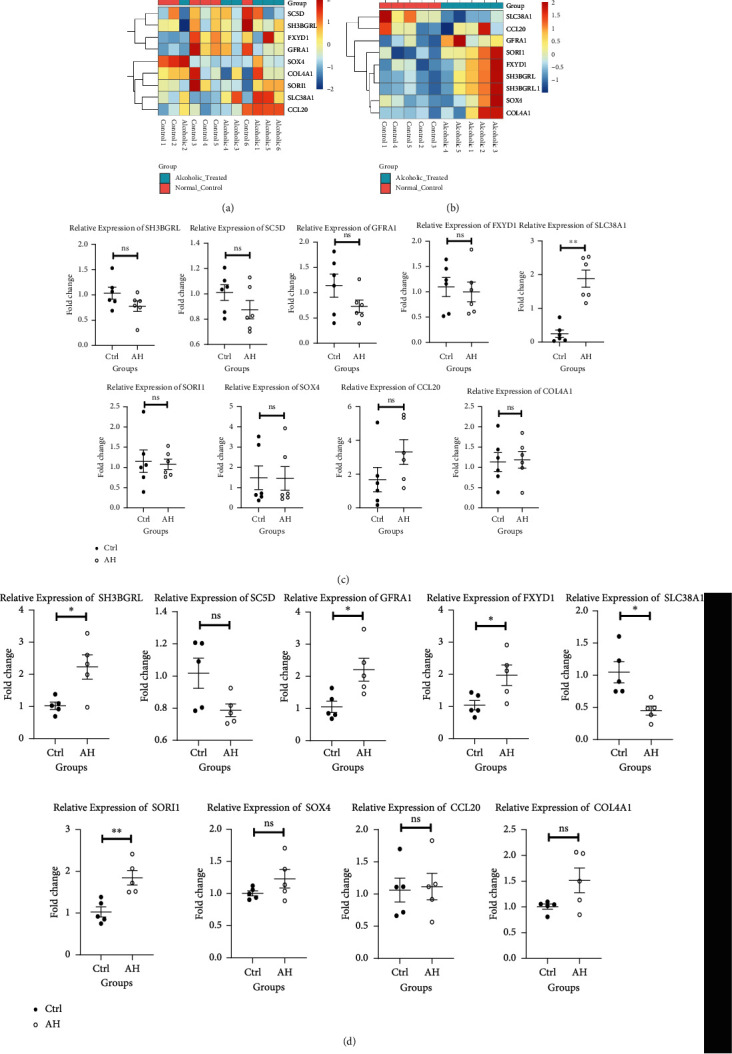
Comparing the levels of different target genes. (a) The heatmap of target gene expression between normal and ASH tissues in animal models. (b) The heatmap of target gene expression between normal and alcohol-stimulated cellular models. (c) Relative target gene levels, using RT-qPCR in animal models. (d) Relative target gene levels, using RT-qPCR in cellular models. ^∗^*P* < 0.05, ^∗∗^*P* < 0.01.

Based on our RT-qPCR results, SLC38A1 was highly expressed in the ASH model group, whereas it was scarcely expressed in cellular models (Figure 9).

### 3.9. Analysis of the SLC38A1 Protein Expression in HCC

**Figure 10:**
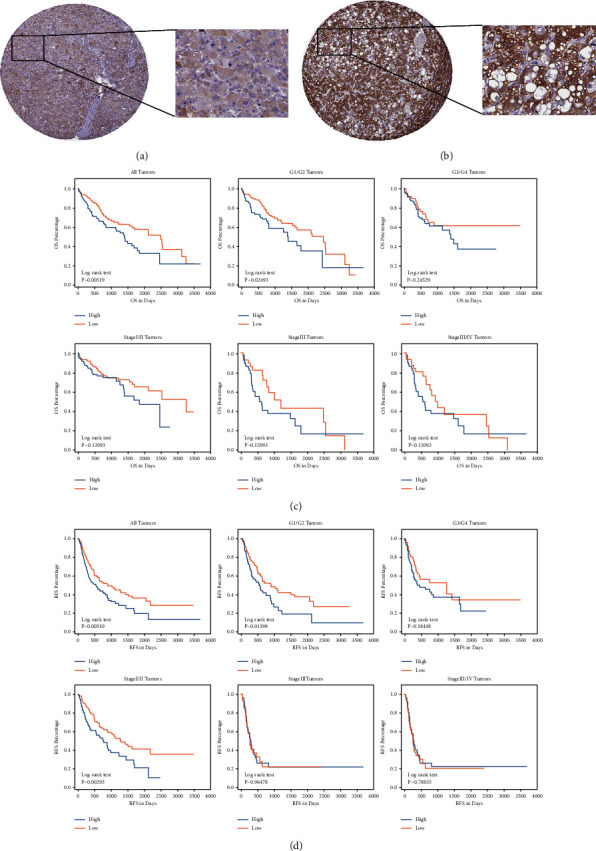
Analysis of the SLC38A1 protein expression in HCC via IHC-based TMA. (a) The SLC38A1 protein expression in HCC specimens without steatosis. (b) The SLC38A1 protein expression in HCC specimens with steatosis. (c) The association between the SLC38A1 protein expression (high or low) and OS rate in HCC patients. (d) The association between the SLC38A1 protein expression (high or low) and RFS rate in HCC patients. OS: overall survival; RFS: relapse free survival.

The IHC-based THPA database was employed for the assessment of SLC38A1 protein expression in HCC tissues. The SLC38A1 protein displayed an elevated expression in HCC tissues with steatosis (Figure 10(a) and 10(b)). OS and RFS were drastically reduced in the enhanced SLC38A1 expression group, as opposed to the reduced SLC38A1 expression group. Given these evidences, the elevated SLC38A1 expression may be a predictor of a worse prognosis in ASH patients (Figure 10(c) and 10(d)).

## 4. Discussion

ASH is usually asymptomatic in the early stage and is already advanced at the time of diagnosis. With a rise in global alcohol abuse, ASH is among the most significant diseases threatening the world now. HCC is one of the final outcomes of multiple liver diseases, but at present, the HCC screening mostly focuses on liver cancer development. Till now, there is no effective biomarker that predicts the prognosis and recurrence of liver cancer. Meanwhile, there are limited studies examining the prognosis and recurrence of HCC patients complicated with ASH.

In this study, the GSE59492, GSE28619, and GSE155907 datasets were obtained from the GEO database. Using DE analysis of GSE59492, we obtained 11 upregulated and 8 downregulated DE miRNAs. Then, utilizing enrichment analysis, we revealed that these DE miRNAs may be related to biological functions like cell proliferation, aging, and oxidative stress, as well as diseases, such as carcinoma, cervical, type 2 diabetes melilites, and osteosarcoma. Among these associations, oxidative stress was closely related to ASH. These DE miRNAs may also play a role in ASH regulation. Therefore, we designed an experiment to test our hypothesis.

The microRNA 182-3P, microRNA 214-5p, microRNA 432, and microRNA 21a-3P expressions were increased in the ASH animal model, compared to the normal group, and the differences were statistically significant. However, in the cell experiment, the expressions of these miRNA were diminished, and the differences were also statistically significant. The animal model was treated with alcohol for 4 weeks. Compared to the cell experiment, the time and intensity of alcohol on liver cells were different. It is possible that the difference in the miRNA expression profile may be the result of self-regulation within animal hepatocytes. In any case, using both cellular and animal models, we demonstrated that alcohol influences the expressions of microRNAs 182-3p, 214-5p, and 432 and 21a-3P in hepatocytes.

We also selected DE genes from the mRNA datasets. A total of 1064 and 2071 DE genes were screened from the GSE28619 and GSE155907 datasets. Next, we obtained the intersection via generation of the Venn diagram. Finally, we obtained 222 co highly expressed genes and 291 co scarcely expressed genes.

One aspect of the miRNAs' biological function was the regulation of target gene mRNA expression in cells. We estimated the target genes of various DE miRNAs and compared this data against the list of DEGs we obtained from the gene expression dataset. Based on the GO analysis of DEGs, we selected some target DEGs for verification *in vivo* and *in vitro*. We discovered that the SLC38A1 expression was markedly enhanced in the animal model and drastically reduced in the cell model, and the differences were statistically significant. SLC38A1 is an amino acid transporter, which is ubiquitous in tumor tissues, where it stimulates tumor cell proliferation, invasion, and migration [22, 23]. However, the relationship between SLC38A1 and prognosis and/or recurrence of liver cancer is yet unreported. In addition, there are no reported investigations on the association between SLC38A1 and the prognosis of liver cancer patients with ASH. Our analysis of the THPA database revealed that the SLC38A levels enhanced markedly in patients with fatty liver disease. Moreover, we revealed that the OS and RFS were drastically diminished in the elevated SLC38A1 expression group versus the reduced SLC38A1 expression group, and differences were statistically significant. In summary, based on our DE profile datasets of ASH, and *in vivo* and *in vitro* data, we drew the following conclusions: (1) ASH results in the high expression of SLC38A1 and miRNA-432 in the liver. (2) High levels of hepatic SLC38A1 in patients with liver cancer cause lower OS and RFS. (3) Liver cancer patients with ASH may also exhibit reduced OS and RFS. (4) miRNA-432 and SLC38A1 exhibited the same expression pattern in our experiment. As such, they may serve as a pair of predictive biomarkers that predict the prognosis and recurrence of HCC complicated with ASH.

In our research, analysis of our cellular and animal models yielded opposite conclusions, and both were statistically significant. This does not necessarily mean that the results were contradictory to one another but provided direction for additional investigations.

As mentioned previously, the time of alcohol stimulation of hepatocytes was different in our cellular and animal models. In fact, in our cellular model, the stimulation was over a short period, whereas in our animal model, it was over a long period of time. Based on this evidence, when cells were stimulated with alcohol, the initial reaction is the downregulation of SLC38A1 and miRNA-432 levels. However, with prolonged alcohol stimulation, the SLC38A1 and miRNA-432 levels in the animal models increased. The underlying mechanism behind this process requires further investigation. In addition, we identified SLC38A1 as the target gene of miRNA-432 and demonstrated that both SLC38A1 and miRNA-432 displayed a similar expression pattern *in vivo* and *in vitro*. Therefore, whether this exists a relationship of mutual regulation or a more complex regulatory mechanism remains to be determined. Overall, a dynamical study, which was aimed at exploring the role of miRNA-432 and SLC38A1 in evaluating OS and RFS in patients with HCC, needs to be performed, followed by potential predictive biomarkers that predict the prognosis and recurrence of HCC complicated with ASH.

## 5. Conclusions

We found that miRNA-432 and SLC38A1 had the potential to be a new pair of noninvasive indicators for evaluating OS and RFS in patients with HCC. High levels of hepatic SLC38A1 in patients with liver cancer cause lower OS and RFS. Through the bioinformatic analysis and experimental verification, we speculate these genetic elements might be strong candidates for use as potential predictive biomarkers that predict the prognosis and recurrence of HCC complicated with ASH.

## Figures and Tables

**Table 1:** The primer sequences of genes in RT-qPCR assay.

miRNA/gene name	Species	Forward primer (5′- >3′)	Reverse primer (5′- >3′)
miR-432	Mouse	CGGGCTCTTGGAGTAGATCAG	CAGCCACAAAAGAGCACAAT
miR-21a-3P	Mouse	CGGGCCAACAGCAGTCGATG	CAGCCACAAAAGAGCACAAT
miR-21a-5P	Mouse	CGGGCTAGCTTATCAGACTG	CAGCCACAAAAGAGCACAAT
miR-214-5P	Mouse	CGGGCTGCCTGTCTACACTT	CAGCCACAAAAGAGCACAAT
miR-182-3P	Mouse	CGGGCGTGGTTCTAGACTT	CAGCCACAAAAGAGCACAAT
miR-132-5P	Mouse	CGGGCAACCGTGGCTTTCGA	CAGCCACAAAAGAGCACAAT
U6	Mouse	GCTTCGGCAGCACATATACTAAAAT	CGCTTCACGAATTTGCGTGTCAT
SOX4	Mouse	GACAGCGACAAGATTCCGTTC	GTTGCCCGACTTCACCTTC
SLC38A1	Mouse	GTCAGCAACGACTCTAATGACTT	GGAATATACTCGTCGCATTTCCT
SORI1	Mouse	CCCGGACTTCATCGCCAAG	AGGACGAGAATAACCCCAGTG
COL4A1	Mouse	TCCGGGAGAGATTGGTTTCC	CTGGCCTATAAGCCCTGGT
CCL20	Mouse	GCCTCTCGTACATACAGACGC	CCAGTTCTGCTTTGGATCAGC
SH3BGRL	Mouse	CTGGCTCTACGGCGATTAAGA	TCTCTCATCCACTTCCGATTCTC
SC5D	Mouse	GGGGTTACAGCAAACTCTACG	GGTGCAGGCCCCTATGAAT
GFRA1	Mouse	GCACCAAGTACCGCACACT	GCGGCAGTTGTAGAGAGACTTC
FXYD1	Mouse	TCCATTCACCTACGATTACCACA	GAATTTGCATCGACATCTCTTGC
GAPDH	Mouse	GGACCTGACCTGCCGTCTAG	GTAGCCCAGGATGCCCTTGA

**Table 2:** A summary of DE miRNAs.

miRNA name	Regulation	logFC	adj.P.Val
hsa-mir-182	Up	2.432051282	0.0113447
hsa-mir-4521	Up	2.023333333	0.004476137
hsa-mir-21	Up	2.011666667	0.000986467
hsa-mir-503	Up	1.970641026	0.0029357
hsa-mir-127	Up	1.863846154	2.00E-05
hsa-mir-214	Up	1.816025641	2.04E-06
hsa-mir-146b	Up	1.78974359	2.23E-05
hsa-mir-132	Up	1.681923077	3.37E-05
hsa-mir-224	Up	1.630641026	0.000863925
hsa-mir-3178	Up	1.61974359	0.001838654
hsa-mir-432	Up	1.585384615	0.002197221
hsa-mir-29c	Down	-1.557948718	0.000527905
hsa-mir-20b	Down	-1.605641026	0.003485341
hsa-mir-15a	Down	-1.727307692	0.000934698
hsa-mir-146a	Down	-1.736538462	0.004370319
hsa-mir-422a	Down	-1.790512821	0.0053786
hsa-mir-451a	Down	-1.930512821	0.010604916
hsa-mir-30e	Down	-2.644871795	2.18E-05
hsa-mir-148a	Down	-2.807948718	1.03E-05

**Table 3:** List of consistent DEGs.

Regulation	DEGs
Up (*n* = 222)	PNMA1,MUM1L1,PFKP,TMSB10,MMP7,ARRDC2,ARL2,SUSD2,ANXA3,COL1A1,SEL1L3,TM4SF4,F2RL1,SORT1,FNDC1,C12orf75,FAM213B,RGS4,LDHB,BAMBI,BACE2,PDP1,VTCN1,GSTP1,ANXA2P2,ATP1A1,CD151,SOX9,C1orf198,PDGFD,SLC51B,BEX2,LXN,SCRN1,TUBA1A,COL5A2,THY1,HSPA2,KRT8,SPP1,LGALS4,H2AFY2,L18,ITGA6,CTSK,SULF2,UBE2Q2,CLDN10,PLCXD3,FMOD,PPDPF,PODXL,GPX2ITGA2,TIMP1,CLIC1,CA12,STK39,EPCAM,TRIM47,TP53I3,BSG,ADAMTS12,EFEMP1,ASPN,EEF1A2,ANXA2,CCDC102B,PDZK1IP1,COL4A2,LUM,MYL12B,F13A1,SLC44A3,TREM2,ITGBL1,SRPX2,LGALS3,SLC22A15,PDGFA,DBNDD1,TACSTD2,TNFRSF21,AKR1B1,HIST1H2AC,ARPC1B,KRT18,RAB11FIP1,LRRC1,KRT19,CXCL10,COL4A1,RNASE1,VCAN,TM4SF1,SH3BGRL3,PTGFRN,LPL,RARRES1,FAT1,CSTB,APOA4,PLVAP,SLIT2,GAPDH,HIST1H1C,STMN2,LBH,COL1A2,PROCR,HGF,CYP1B1,ACKR3,WLS,SPINK1,HSPB8,LAMA2,S100P,GOLM1,SOX4,MFSD6,EPDR1,RAB8B,GJA1,GPNMB,KIAA1522,AKR1B10,DEFB1,SQSTM1,FAM150B,PLP2,TAGLN2,ANXA13,LGALS1,COL15A1,CRYAB,NCF2,PPAP2C,IGFBP7,NCEH1DSG2,SLC12A2,CLDN7,CD58,TUBA1B,ABCB1,FAM171A1,ELOVL7,C4orf48,LTBP2,CHST4,LGALS3BP,ITGAV,CAPG,CCL20,ROBO1,ADAM9,TPM1,CIDEC,MCAM,S100A6,TES,FBN1,DCDC2,C7,ALDOA,LASP1,TNFRSF12A,LAMC1,S100A11,ANXA5,MVP,COL3A1,MOXD1,TMEM45B,MARCKS,SERPINH1,CD59,FAP,SGK223,CRYAA,SERPINE2,WBP5,LAMB1,ENPP5NQO1,TSPOCKLF,RAB3B,CCDC80,GPR34,ENDOD1,CXCL9,AEBP1,SPINT1,THBS2,CXCL1,PAPSS1,BICC1,PROM1,S100A10,AQP1,MAP1B,SLC38A1,FABP4,CXCL6,COL6A3,GMNN,LCN2,SPARC,COL5A1,PMEPA1,CTHRC1,GEM,ANXA4,TESC,TRNP1,SPINT2,FLNA,CLIC6,KRT23,FBLN5

Down (*n* = 291)	BBOX1,CYP26A1,XDH,SALL1,CYP39A1,HMGCS2,EPB41L4B,RGN,ZG16,AQP9,RCL1,SORL1,ACSM3,CYP2C19,TMEM52,ZNF385B,ETNK2,ALDH2,NTHL1,BCHE,CPEB3,ADI1,ATP11C,ALAD,TSPAN7,IDO2,CYP1A1,EBPL,SDC2,SLC38A4,PALM2,L6R,NFIL3,HLF,CDHR5,LC39A5,CYP7A1,SRD5A2,ADRA1A,PBLD,PXMP2,NR1I3,KLKB1,ABCC6P1,LINC01093,SLC25A18,ALDH6A1,IGSF9,HAGH,NECAB2,FST,ARHGEF26,COQ10A,PGM1,RCAN1,DCAF11,PHGDH,AQP11,SH3BGRL2,CBS,ADH1C,AZGP1P1,SLC16A2,SC5D,CLEC4M,CYP4F12,SLCO1B1,GCH1,DMGDH,CSRNP1,MME,ALAS1,APOF,GCDH,GADD45B,TMCO6,DBHAS1,ALDH1L1,CCS,PNPLA3,MT1E,CES3,ANXA10,TTC36,FNIP2,AASS,SLC6A1,ADHFE1,GYS2,ADORA2AAS1,KLHL15,CXCL2,GPR128,LYVE1,ASPG,SRD5A1,ASPDH,UPB1,CLDN14,MUT,AGL,F13B,AKR1D1,GNE,CTPS1,NSUN6,CYP3A43,KCNJ3,RALGPS2,GSDMB,ADH4,LINC01018,C7orf55,ID2,CETP,GAS2,DCPS,IL1RN,HERC5,HAO2,MT1F,DAK,KCNN2,HFE2,LIPC,GSTZ1,CNDP1,ACACB,PPP1R1A,RORC,FXYD1,GFRA1,MARCO,AGXT,LCAT,AIG1,DHRS1,RAD54L2,IGFBP3,HAMP,N4BP2L1,PCOLCE2,ASB9,HGFAC,HSD17B14,TMEM56,PZP,AOX1,KCNJ8,SLC19A2,OAT,AGPAT9,COBLL1,CXCL14,CAPN3,DEPDC7,SLCO1B3,BCO2,C3P1,FOLH1B,PON1,DNASE1L3,PKLR,IGFALS,VSNL1,PRRG4,SLC47A1,MT1M,TMPRSS6,CYP4F3,KHK,ECM1,ABHD15,FCN2,LEPR,CYP2A6,PGLYRP2,TENM1,SLC22A1,IL13RA2,HPR,ANO1,APOA5,CFHR4,SLC1A2,ASPA,MAN1C1,ST3GAL6,DPYS,TSPAN33,CTH,IL1RAP,ALDH7A1,TRIB1,RDH16,PER2,DSG1,ADRB2,HPGD,DNAJC12,GCHFR,CYP4A11,MT1X,SERPINF2,CUX2,GLYCTK,CTNNA3,AADAT,GPR88,CHAD,ACADS,EPHX2,FAM151A,PLIN1,LINC00844,DIRAS3,ETNPPL,SULT1E1,GLUD1,ABCG2,GADD45G,DNMT3L,FITM1,CYP4X1,CYP1A2,ACADSB,HGD,CDO1,LIME1,CD5L,LPA,EXPH5,PSAT1,CYP27A1,GNMT,NR5A2,AVPR1A,DGAT2,FMO5,THNSL1,SMOC1GSTA3,SORD,PPAP2B,SMIM14,GLYAT,ADH1A,ANGPTL6,SCML1,SSTR1,PAIP2B,MAT1A,CMBL,MPDZ,IYD,SOCS2,SLC13A5,CENPV,ACMSD,HAAO,LY6E,GCAT,ENPP1,GBA3,ECHDC2,CLEC4G,GPR125,MLXIPL,CFHR3,ADH6,AMT,PVRL3,VIPR1,PLAC8,GPT2,CFP,NR1I2,FTCD,RELN,UAP1,DHODH,PHYHD1,AR,ZGPAT,CISH,OGDHL,SLCO4C1,SLC27A5,ACSM2B,ATF7IP2,BCKDHB

Note: DEGs: Differentially expressed genes.

## Data Availability

The data will be available upon reasonable request.
